# Lidocaine combined with magnesium sulfate preserved hemodynamic stability during general anesthesia without prolonging neuromuscular blockade: a randomized, double-blind, controlled trial

**DOI:** 10.1186/s12871-021-01311-y

**Published:** 2021-03-27

**Authors:** Waynice N Paula-Garcia, Gustavo H Oliveira-Paula, Hans Donald de Boer, Luis Vicente Garcia

**Affiliations:** 1 Department of Orthopedics and Anesthesiology, Faculty of Medicine of Ribeirao Preto, University of São Paulo, Ribeirão Preto, SP, 14049900 Brazil; 2 Albert Einstein College of Medicine, New York 1300 Morris Park Ave, The Bronx, NY 10461 USA; 3 Department of Anesthesiology, Pain Medicine and Procedural Sedation and Analgesia, Martini General Hospital Groningen, van Swietenplein 1, 9728 NT Groningen, The Netherlands

**Keywords:** Lidocaine, Magnesium sulfate, Neuromuscular blockade, General anesthesia, Hemodynamic parameters

## Abstract

**Background:**

Lidocaine and magnesium sulfate have become increasingly utilized in general anesthesia. The present study evaluated the effects of these drugs, isolated or combined, on hemodynamic parameters as well as on the cisatracurium-induced neuromuscular blockade (NMB).

**Methods:**

At a university hospital, 64 patients, ASA physical status I and II, undergoing elective surgery with similar pain stimuli were randomly assigned to four groups. Patients received a bolus of lidocaine and magnesium sulfate before the tracheal intubation and a continuous infusion during the operation as follows: 3 mg.kg^− 1^ and 3 mg.kg^− 1^.h^− 1^ (lidocaine - L group), 40 mg.kg^− 1^ and 20 mg.kg^− 1^.h^− 1^ (magnesium - M group), equal doses of both drugs (magnesium plus lidocaine - ML group), and an equivalent volume of isotonic solution (control - C group). Hemodynamic parameters and neuromuscular blockade features were continuously monitored until spontaneous recovery of the train of four (TOF) ratio (TOFR > 0.9).

**Results:**

The magnesium sulfate significantly prolonged all NMB recovery features, without changing the speed of onset of cisatracurium. The addition of lidocaine to Magnesium Sulfate did not influence the cisatracurium neuromuscular blockade. A similar finding was observed when this drug was used alone, with a significantly smaller fluctuation of mean arterial pressure (MAP) and heart rate (HR) measures during anesthesia induction and maintenance. Interestingly, the percentage of patients who achieved a TOFR of 90% without reaching T1–95% was higher in the M and ML groups. Than in the C and L groups. There were no adverse events reported in this study.

**Conclusion:**

Intravenous lidocaine plays a significant role in the hemodynamic stability of patients under general anesthesia without exerting any additional impact on the NMB, even combined with magnesium sulfate. Aside from prolonging all NMB recovery characteristics without altering the onset speed, magnesium sulfate enhances the TOF recovery rate without T1 recovery. Our findings may aid clinical decisions involving the use of these drugs by encouraging their association in multimodal anesthesia or other therapeutic purposes.

**Trial registration:**

NCT02483611 (registration date: 06-29-2015).

**Supplementary Information:**

The online version contains supplementary material available at 10.1186/s12871-021-01311-y.

## Background

Anesthetic additive drugs, such as lidocaine and magnesium sulfate (MS), have become increasingly utilized, either alone or in combination, in general anesthesia for postoperative pain reduction, achievement of reduced and more balanced anesthetic doses, hemodynamic stabilization, and improvement of surgical conditions [1, 2]. Opioid-sparing or opioid-free anesthesia is a relatively new strategy that is increasingly being used in daily anesthesia practice [3]. A combination of lidocaine and magnesium sulfate in a multimodal opioid-sparing or even opioid-free anesthetic approach may reduce or eliminate the use of opioids in the perioperative period [4, 5]. Drugs such as lidocaine and magnesium sulfate are frequently used in combination with neuromuscular blocking agents (NMBAs) [6], the latter of which may contribute to residual neuromuscular blockade (NMB). It is a well-known [7] and ongoing problem [8] that NMBAs have the inherent risk of residual paralysis [9], even when used alone [10]. Furthermore, residual paralysis is most likely associated with postoperative pulmonary complications, which has also been well known for many years [11] but has still not been resolved [12].

Magnesium sulfate infusion administered before anesthesia has been found to increase the speed of onset of a rocuronium-, cisatracurium- or vecuronium-induced NMB without necessarily enhancing its duration [13, 14]. Furthermore, magnesium sulfate infusion re-establishes a clinically relevant degree of muscle paralysis in patients who have recovered from paralysis and causes a significant, prolonged NMB when induced by a single dose of the neuromuscular-blocking drug rocuronium [15].

The effects of lidocaine on NMB are still under debate. Previous studies have shown that local anesthetics, such as lidocaine, interact with NMBAs [16–18]. More recent studies evaluating the clinical effects of lidocaine, at lower dosages, on the NMBAs cisatracurium and rocuronium have demonstrated no changes in the recovery of NMB characteristics or speed of onset [19–21].

Considering the growing perioperative clinical applications of both lidocaine and magnesium sulfate, the possibility of using these drugs in combination increases. A combination of these drugs may not only be beneficial for surgical patients regarding opioid-sparing effects but may also influence NMB characteristics and promote changes in hemodynamic parameters. The main objective was to evaluate whether the use of additional lidocaine could influence the NMB enhancement. Thus, this study’s primary endpoint was the time at which spontaneous recovery of a train-of-four (TOF) ratio of 90% was achieved (complete duration). The secondary endpoints were other NMB characteristics (onset time, duration 25, duration 95) and hemodynamic parameters.

## Methods

In this prospective, randomized, double-blind, controlled trial, sixty-six patients [American Society of Anesthesiologists (ASA) physical status I to II, aged 18 to 60 years] were recruited who were scheduled for surgery (estimated surgical time greater than 90 min, with a similar pain stimulus and no need for a continuous neuromuscular block during the surgical procedure). The exclusion criteria were patients with diseases or on medications known to interfere with neuromuscular transmission, hepatic or renal dysfunction, electrolyte abnormalities (hypokalemia, hypocalcemia and hypermagnesemia, which can potentiate blockade), allergy to drugs used in the study, a body mass index < 18 or > 29 kg.m^− 2^, and expected difficulties during mask ventilation or intubation (mouth opening and head and neck movement limitations, a short thyromental and sternomental distance, and a history of difficult intubation), as well as patients who were pregnant or breastfeeding.

**Fig. 1:**
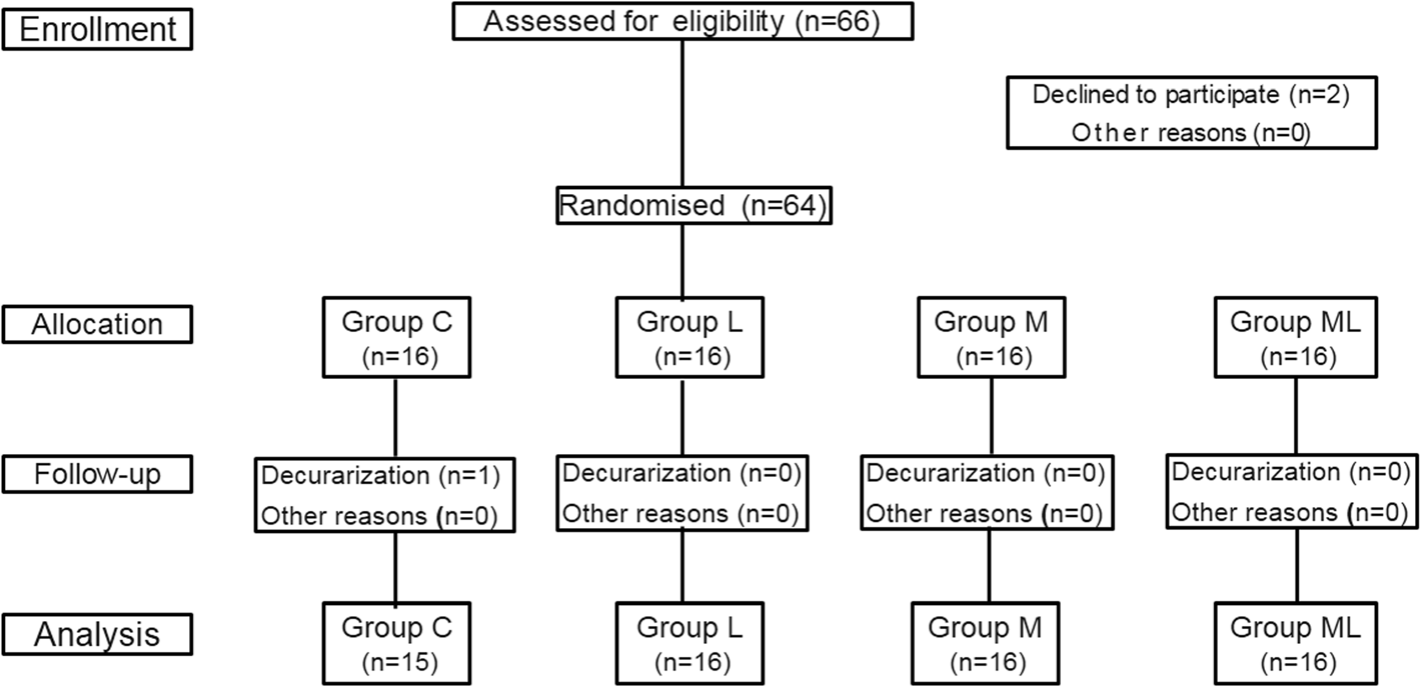
Flow chart of patient participation. C: control group; M: magnesium sulfate group; ML: magnesium sulfate combined with lidocaine group

The patients were randomly and equally allocated into four groups (Fig. 1). Computer-generated randomization was performed, and allocation was concealed with sequentially, numbered, sealed, opaque envelopes. The seal of the envelope was broken before the induction of general anesthesia by trained study personnel not involved in the data collection. Throughout the perioperative period, care providers, patients, and research team members were blinded to the group assignment. The L group received 3 mg.kg^− 1^ lidocaine as an IV bolus before the induction of anesthesia and 3 mg.kg^− 1^.h^− 1^ lidocaine via IV continuous infusion during the operative period, and the M group received 40 mg.kg^− 1^ magnesium sulfate as an IV bolus before the induction of anesthesia and 20 mg.kg^− 1^.h^− 1^ magnesium sulfate via IV continuous infusion during the operative period. The ML group received equal doses of magnesium sulfate combined with lidocaine at the same conditions during the operative period, and the control group received an equivalent volume of isotonic solution.

Patients were monitored using electrocardiography, noninvasive blood pressure, pulse oximetry, capnography, and body temperature (Dixtal Biomedical Industry e commerce, Brazil). Total intravenous anesthesia (TIVA) was standardized for all patients and performed without the use of benzodiazepines, using a propofol target dose (plasma targeting, Injectomat TIVA Agilia, Brazil) of 4 μg·mL^− 1^ and a remifentanil infusion of 0.5 mg·kg^− 1^·min^− 1^. After induction, the propofol infusion target was decreased to 2.5 μg·mL^− 1^, and infusion of remifentanil was adjusted to 0.1–0.3 μg·kg^− 1^·min^− 1^ as needed. If systolic arterial pressure (SAP) or heart rate (HR) increased or decreased by > 30% of baseline for > 60 s, remifentanil infusion was respectively increased/decreased at 0.05 μg kg^− 1^ min^− 1^ until achieving the goal value within the range. If necessary, an ephedrine bolus (2.5 to 5 mg) was allowed. Hemodynamic parameters were considered stable when blood pressure and HR were within 20% of baseline values.

After induction of anesthesia and loss of consciousness, neuromuscular function was assessed by monitoring the adductor pollicis muscle via acceleromyography (AMG) with a TOF-Watch SX device (Organon Ireland Ltd., a subsidiary of Merck & Co., Inc., Swords, Co., Dublin, Ireland) according to the neuromuscular research consensus [22].

The monitoring system was positioned on the side opposite to the blood pressure cuff and IV line. Pediatric surface electrodes (Red Dot®, 3 M Health Care, Neuss, Germany) were placed on cleaned skin over the ulnar nerve on the volar side of the wrist. The transducer’s position was secured by placing the thumb in a hand adapter, and a temperature sensor was fixed at the distal end of the forearm. TOF stimulation tracing was stabilized by administering TOF repetitive stimulation for 1 min, followed by 5 s of 50-Hz tetanus stimulation and another period of repetitive TOF stimulation for 3–4 min. The CAL 2 mode was used to determine the supramaximal threshold and to calibrate the transducer of the accelerometer. After device calibration and stabilization, the mean of three TOF values was recorded in each patient and used as a reference. Complete recovery was assumed when the TOF ratio (TOFR) reached 90% of the preoperatively defined reference value (normalization). The same procedure was performed for the T1 measurements, assuming T1 response recovery as 95% of its initial value. Then, bolus doses of solutions were administered to assigned groups over 5 min. Subsequently, single twitches were monitored to measure the onset time of cisatracurium (0.15 mg.kg^− 1^ administered over 5 s; time point zero). Tracheal intubation was performed when TOFR reached zero. Values of T1, T2, T3, T4, and TOFR (T4/T1); twitch responses in relation to the reference twitch response given in %; and skin temperature were also recorded. Patients were monitored until they achieved spontaneous recovery from NMB (TOFR = 0.9). No additional cisatracurium injections were permitted. After measuring the onset time, the stimulation mode was changed to TOF (2 Hz, stimulus duration of 200 μs, square wave, 15-s intervals).

All neuromuscular monitoring data were transferred in real-time and stored on a laptop using the TOF-Watch SX monitor computer program (version 2.5.INT; Organon Ltd., Dublin, Ireland).

The following variables were measured:

(1) onset time 5 (time [min] from injection to T1 response < 5% of T0); (2) duration 25 (time [min] to spontaneous recovery of T1 to 25%); (3) recovery index (time [min] between T1 = 25% and T1 = 75%); (4) duration 95 (time [min] from injection to spontaneous recovery of T1 to 95%); and (5) complete duration (time [min] from injection to spontaneous recovery of TOFR> 0.9).

Hemodynamic parameters (systolic, diastolic, mean blood pressure and heart rate) were recorded and annotated at various times: M1- when the patient arrived in the operating room; M2- immediately before induction of anesthesia; M3- before the infusion of the tested solutions (saline, magnesium sulfate or magnesium sulfate associated with lidocaine); M4- five minutes after M3 (end of the infusion loading dose of test solutions); M5- immediately before intubation; M6- one minute after tracheal intubation; and M7- every fifteen minutes until the end of the study. Heating elements were used to maintain the skin and central temperatures above 32 and 36 °C, respectively. All unexpected events that occurred during the study were recorded as adverse effects.

The Shapiro-Wilk test was used to assess normality. The clinical and demographic characteristics are expressed as the means ± SD or medians (IQR [range]) and were compared by analysis of variance, the Kruskal-Wallis test, or the chi-square test where appropriate. The area under the curve (AUC) was assessed to compare the hemodynamic responses between the study groups [23]. The pharmacodynamic variables (i.e., speed of onset, clinical duration, recovery rate, and complete duration) are represented as box-and-whisker plots showing the range, quartiles, and medians. The AUCs of the changes in mean arterial pressure (MAP) and HR are expressed as 95% confidence intervals (normally distributed data). The pharmacodynamic variables were compared between the groups via the Kruskal-Wallis test, followed by Dunn’s multiple comparison test. The AUCs of the changes in MAP and HR were compared between the groups by one-way ANOVA followed by the Tukey multiple comparison test. Both multiple comparison tests were used to control the type I error at 5%. The percentages of patients who achieved a TOFR of 90% without reaching 95% recovery of the first twitch (T1) response were compared between the groups by the chi-square test. A *p*-value < 0.05 was considered statistically significant for all outcome variables.

The primary endpoint of the present study was the time at which spontaneous recovery of a train-of-four (TOF) ratio of 90% was achieved (complete duration). Therefore, for the sample size calculation, we considered a previous study showing that magnesium sulfate prolonged the complete duration of rocuronium-induced NMB [13]. Having chosen a significance level of 5% and a power of 80%, we applied the Satterthwaite’s approximation [24]. The result revealed *N* = 14 patients per group, and we decided to randomized 16 patients in each group to allow for drop-outs. The statistical analysis plan has been added as a supplement file.

## Results

Between 2015 and 2018, 64 patients were recruited and randomized in this study. The patient characteristics are shown in Table 1. The vast majority of patients were ASA1, who underwent rhinoplasty and reductive mastopexy. There was no significant difference in the baseline variables between the groups. After data collection, one patient was excluded from the control group because her surgical procedure was completed in less than 90 min (Fig. 1).

**Table 1:** Clinical and demographic characteristics of the patients

	C (***n*** = 16)	L (n = 16)	M (n = 16)	ML (n = 16)	*p* value
Age (years)	36 + 11	34 + 9	34 + 11	32 + 9	NS
Gender(F/M)	7/8	8/8	8/8	8/8	NS
ASA PS I/II	14/1	14/2	14/2	13/3	NS
BMI(Kg/m^2^)	24.1 + 3.3	24.9 + 3.4	25.7 + 3.4	23.2 + 2.6	NS

*ASA PS* American Society of Anesthesiologists phyical status, *BMI* body mass index, *C* control group, *L* lidocaine group, *M* magnesium sulfate group, *ML* magnesium sulfate plus lidocaine group. Values are the mean + S.D.

Magnesium sulfate significantly prolonged all NMB recovery features, without changing the speed of onset of cisatracurium (Table 2). The addition of lidocaine to MS did not influence the cisatracurium NMB. Similar findings were observed when this drug was used alone.

**Table 2:** Neuromuscular blockade recovery characteristics

	Control	Lidocaine	Magnesium	Magnesium + Lidocaine	***p***
**onset time 5**	144 (120–165[min])	135 (117–155[min])	145 (116–177[min])	138 (109–168[min])	> 0,05
**duration 25**	64 (57–70[min])	69 (63–79[min])	82 (76–91[min])	85 (82–88[min])	< 0,0001
**recovery index**	14 (14–16[min])	16 (11–19[min])	24 (16–30[min])	20 (18–26[min])	< 0,0001
**duration 95**	87 (66–90[min])	88 (81–101[min])	109 (104–126[min])	113 (95–117[min])	< 0,0001
**complete duration**	89 (76–99[min])	104 (93–107[min])	119 (110–129[min])	123 (111–140[min])	< 0,0001

Values are medians (Interquartile range [min])

**Fig. 2:**
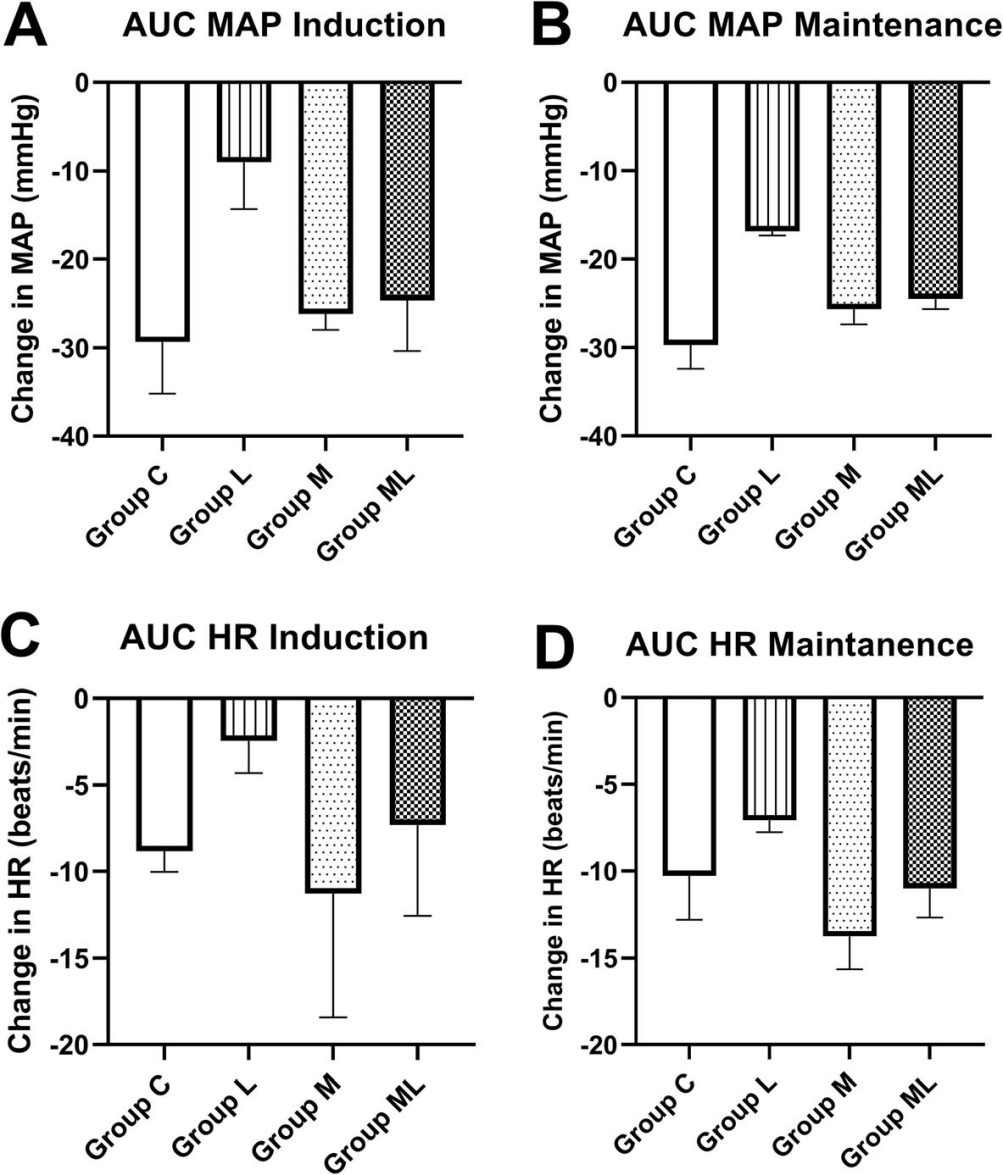
Area under the curve (AUC) of hemodynamic parameters. **a** AUC of the mean arterial pressure (MAP) in the induction period. **b** AUC of the MAP during the maintenance period. **c** AUC of heart rate (HR) in the induction period. **d** AUC of HR during the maintenance period. C: control group; M: magnesium sulfate group; ML: magnesium sulfate combined with lidocaine group. Values are the mean change and 95% CI

The hemodynamic parameters among the study groups, evaluated by the AUCs for changes in MAP and HR at the six times points during anesthesia induction, are shown in Fig. 2 (a-d). The lidocaine group presented a significantly smaller fluctuation of MAP and HR measures during anesthesia induction (total AUC [95% confidence interval; p]: MAP- L Group, 18.4 [0.0–41.3], compared with the C group, 59.9 [34.7.0–88.1], *p* < 0.0001; compared with the M group, 52.2 [32.3–72.0], *p* < 0.0001; compared with the ML Group, 50.3 [32.3–68.4], p < 0.0001, and HR- L Group, 4.9 [0.0–15.3] compared with the C group, 17.9 [0.0–47.4], p < 0.0001; compared with the M group, 23.0 [1.7.-44.4], *p* < 0.001; compared with the ML Group, 14.9 [0.0–37.2], *p* < 0.046). During the maintenance phase of anesthesia, the study groups exhibited a similar behavior (MAP- L Group, 1265 [583.5–1946] compared with the C group, 2256 [1529–2983], *p* < 0.021; compared with the M group, 1891 [1334–2448], p < 0.0001; compared with the ML Group, 1837 [1338–2335], p < 0.0001 and HR- L Group, 533.0 [259.8–806.1] compared with the C group, 795.8 [125.2–1466], *p* < 0.01; compared with the M group, 1035.0 [360.8–1708], p < 0.001; compared with the ML Group, 828.3 [294.0–1363], *p* < 0.0043).

Interestingly, the percentage of patients who achieved a TOFR of 90% without reaching T1–95% was higher in the M and ML groups compared with the C and L groups (50.0, 56.2 and 20.0%, 25.0%, respectively). There were no adverse events reported in this study.

## Discussion

The main findings of the study showed the following: (a) intravenous lidocaine plays a significant role in the hemodynamic stability of patients under general anesthesia, without exerting any additional impact on the NMB even when combined with magnesium sulfate; (b) magnesium sulfate prolonged the time of recovery from NMB in all pharmacodynamic parameters studied; and (c) there were no differences in the speed of onset of NMB between groups.

The concept of multimodal general anesthesia has recently extended the idea of balanced anesthesia, including the use of some other additional drugs such as lidocaine, magnesium sulfate, β–blockers, and α2-agonists, which target different neuroanatomical circuits and multiple neurophysiological mechanisms [25]. The pharmacological explanation of the multimodal general anesthesia approach is based on the firmly established observation that when anesthetic drugs of different mechanisms are combined, they typically interact synergistically [26]. Lidocaine and magnesium sulfate indirectly block sympathetic effects and are well established in opioid-sparing multimodal analgesic strategies [27]. Parenteral magnesium facilitates an NMB by decreasing pre-junctional release of acetylcholine via inhibition of voltage-dependent calcium channels [28]. Typically, magnesium sulfate is administered as a bolus dose of 30–50 mg.kg^− 1^, followed by a maintenance dose of 6–20 mg.kg^− 1^.h^− 1^ [29]. The results of this study were in accordance with previous studies concerning the prolongation of NMB by magnesium [13, 30, 31].

Contradictory findings have been reported concerning whether the NMB onset time was reduced by magnesium sulfate. Preadministration of MS infused over 10 min has been shown to reduce the onset time of rocuronium and vecuronium [30, 32], while a short period of infusion left the neuromuscular blockers’ latency unchanged, in accordance with our findings [33, 34]. These findings possibly reflect differences in NMBA pharmacodynamic properties, and the higher infusion time seemed to prolong the drug’s action.

Interestingly, many patients in magnesium sulfate infusion groups reached 90% of their initial TOF response without recovery of T1 to 95% of its original value. Staals et al [35] reported findings similar to ours after reversing rocuronium-induced NMB with sugammadex. Especially when using AMG devices, a TOFR of 0.9 seems to be associated with insufficient recovery of neuromuscular function [36, 37].

Perioperative IV administration of lidocaine varies among studies based on the dose of lidocaine bolus (1–5 mg kg^− 1^), maintenance infusion (1–6 mg kg^− 1^ h^− 1^) and duration of infusion [2, 38]. However, the use of doses and lidocaine as high as 5 mg.kg.h^− 1^ infused for 6 h is reported without adverse effects [39]. Typically, the lidocaine dose used in studies assessing its impact on NMB [19–21] has been between 1.5 and 2 mg.kg^− 1^ (bolus) and 2 mg.kg^− 1^.h^− 1^ (maintenance), and results similar to ours were reported in these studies. Although lidocaine is widely used and is especially useful as an adjuvant during general anesthesia due to its analgesic and opioid-sparing effects, few studies have systematically assessed the incidence of adverse effects or optimal dose [2]. We have considered using high doses to evaluate possible hemodynamic changes. Some studies have shown some interactions between local anesthetics and NMBAs [16–18, 40]. However, more recently, studies evaluating the clinical effects of lidocaine on cisatracurium- and rocuronium-induced NMB have demonstrated no changes in NMB recovery characteristics or speed of onset periods [19–21]. Corroborating these observations, in the present study, even lidocaine infusion at higher doses did not result in any additional effects on NMB and reduced MAP and HR fluctuations. Importantly, this hemodynamic stability is particularly relevant in specific conditions, such as in intracranial aneurysm management [38].

Surgical patients need to be fully awake in the recovery ward postoperatively, with acceptable pain levels and without respiratory depression, especially for patients with morbid obesity or obstructive sleep apnea [41]. It is also known that opioids present side effects, including postoperative nausea and vomiting, shivering, ileus, and urine retention [42], and can achieve both short-lasting analgesia and long-lasting hyperalgesia due to their upregulation of compensatory pronociceptive pathways [43].

Therefore, opioid-free or opioid-reduced anesthesia procedures are justified and have gained increasing popularity [44, 45]. Given these new situational demands for anesthesia and pain control protocols, evidence that lidocaine can provide hemodynamic stability during anesthesia and that its addition to magnesium sulfate does not add any side effects is valuable. These findings may encourage the use of lidocaine infusion alone or combined with magnesium sulfate in clinical practice for various therapeutic purposes, including opioid-free/sparring anesthesia with or without NMBA. The mechanisms and the precise dosage regimen for the hemodynamic stability provided by lidocaine warrant further research.

Our study has some limitations. The actual plasma concentrations of cisatracurium were not measured. However, we chose this nondepolarizing NMBA because its duration of action has low inter-individual variability. All groups have been treated with remifentanil intraoperatively and, assuming that Mg and lidocaine have analgesic effects, the infusion rate of remifentanil may have not been adjusted accordingly. Therefore, we cannot completely rule out a possible interference of this drug on the hemodynamic results shown in this study.

## Conclusions

Intravenous lidocaine plays a significant role in the hemodynamic stability in adult patients under general anesthesia without exerting any additional impact on the NMB, even when combined with magnesium sulfate. Aside from prolonging all NMB recovery characteristics without altering the onset speed, magnesium sulfate enhances the TOF recovery rate without T1 recovery. Our findings may aid clinical decisions involving the use of these drugs by encouraging their association in multimodal anesthesia or other therapeutic purposes.

## Supplementary Information

**Additional file 1.**

## Data Availability

The datasets generated and analyzed during the current study are not publicly available, as permission from participants to publicity share the dataset has not been obtained. However, these datasets are available from the corresponding author on reasonable request.

## Abbreviations

AMG: Acceleromyography
ASA: American Society of Anesthesiologists
AUC: Area under the curve
HR: Heart rate
MAP: Mean arterial pressure
MBP: Mean blood pressure
MS: Magnesium Sulfate
NMB: Neuromuscular blockade
NMBA: Neuromuscular blocking agent
SAP: Systolic arterial pressure
SD: Standard deviation
TIVA: Total intravenous anesthesia
TOF: Train of four
TOFR: Train of four ratio
