# Supplementary material for: Lidocaine combined with magnesium sulfate preserved hemodynamic stability during general anesthesia without prolonging neuromuscular blockade: a randomized, double-blind, controlled trial
Source: BMC Anesthesiol. 2021 Mar 27;21:91. doi: 10.1186/s12871-021-01311-y (PMC8004390; doi:10.1186/s12871-021-01311-y)
Supplement: Supplementary file 1 — Additional file 1. [file 12871_2021_1311_MOESM1_ESM.pdf]

Statistical Analysis Plan (SAP)

Lidocaine Plus Magnesium Sulfate on Cisatracurium Blockade

Principal Investigator	Waynice Neiva de Paula Garcia, MD, PhD, Assistant Professor of Anesthesiology. Clinical Hospital of Ribeirao Preto - University of Sao Paulo, Ribeirao Preto, Brazil
Protocol identification number	Unique Protocol ID is 5362/2013
ClinicalTrials.gov identifier	NCT02483611
Version	1

Abbreviations

CI	Confidence Interval
DBP	Diastolic Blood Pressure
IQR	Interquartile Range
NMB	Neuromuscular Blockade
RCT	Randomized Clinical Trial
SAP	Statistical Analysis Plan
SBP	Systolic Blood Pressure
SD	Standard Deviation
TOF	Train of Four

Table ofcontents

1. Introduction....4
2. Study design....4
   2.1 Sample size calculation....5
3. Aims and objectives....5
4. Outcomes....5
   4.1 Primary outcome....5
   4.2 Secondary outcomes....5
   4.3 Safety outcomes....6
5. Populations and subgroups to be analyzed....6
   5.1 Populations....6
   5.2 Subgroups....6
6. Analyses....6
   6.1 Primary outcome....7
   6.2 Secondary outcomes....7
7. Missing data....7

1. Introduction

The aim of this project is to evaluate in a single-center randomized clinical trial (RCT) whether the use of additional lidocaine could influence the NMB enhancement. Thus, this study's primary endpoint is the time at which spontaneous recovery of a train-of-four (TOF) ratio of 90% will be achieved (complete duration). The secondary endpoints are other NMB characteristics (onset time, duration 25, duration 95) and hemodynamic parameters.

This statistical analysis plan (SAP) will give more detailed descriptions of the endpoints in the study and the corresponding analyses.

2. Study design

Sixty-four patients [American Society of Anesthesiologists (ASA) physical status I to II, aged 18 to 60 years] who will be scheduled for surgery (estimated surgical time greater than 90 min, with similar pain stimulus and no need for a continuous neuromuscular block during the surgical procedure) will be recruited. The exclusion criteria are patients with diseases or on medications known to interfere with neuromuscular transmission, hepatic or renal dysfunction, electrolyte abnormalities that can potentiate blockade, an allergy to the drugs used in the study, a body mass index <18 or >29 kg.m-2, and those who are expected to have difficulties during mask ventilation or intubation, pregnant or breastfeeding.
The patients will be randomly and equally allocated into four groups (figure 1). The Computer-generated randomization will be stratified by gender to pursue equal distribution of intervention and control group for both male and female subjects, and the allocation concealed with sequentially numbered, sealed, opaque envelopes. The envelope's seal will be broken before the induction of general anesthesia by trained study personnel not involved in the data collection. Throughout the perioperative period, care providers, patients, and research team members will be blinded to the group assignments.

Figure 1 Flowchart of screening and inclusion process

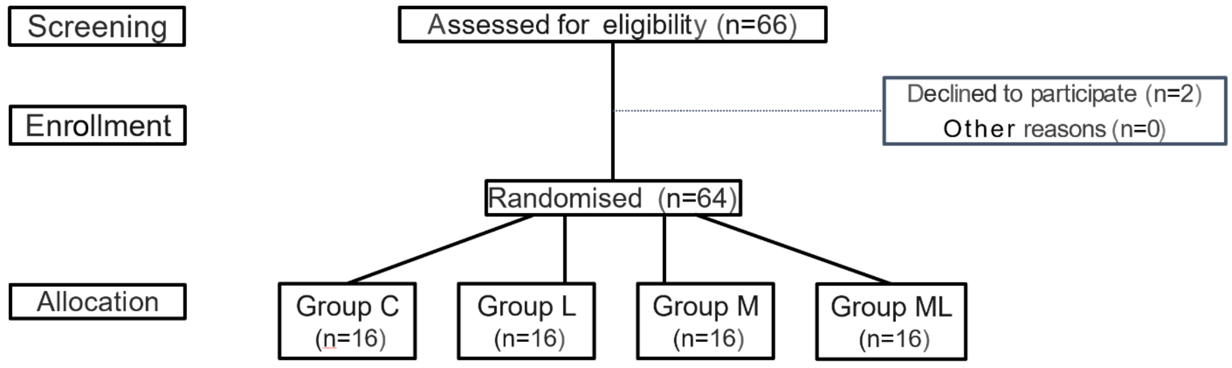

2.1 Sample size calculation

The primary outcome measure for the power calculation is the elapsed time for the TOF (T4/T1) recovery response to 90% of the original value after infusion of cisatracurium. For the sample size calculation, we considered a previous study showing magnesium sulfate prolonged the complete duration of rocuronium-induced NMB (Acta Anaesthesiol Scand 2010, 54(3):299-306). Having chosen a significance level of 5% and a power of 80%, we applied Satterthwaite's approximation (*Stat Med* 2001, 20(7):1089-1095). The result revealed N=14 patients per group, and we decided to randomized 16 patients in each group to allow for drop-outs.

3. Aims and objectives

To investigate the effects of lidocaine associated with magnesium sulfate on the neuromuscular blockade promoted by cisatracurium and evaluate its possible hemodynamic repercussions.

4. Outcomes

This section will present the outcomes investigated to answer the study aims and objectives. The analyses are described in section 6- Analyses.

4.1 Primary outcome

Complete duration (T4/T1=90%): elapsed time for the TOF (T4/T1) recovery response to 90% of the original value after infusion of cisatracurium. This outcome measure will be presented in minutes.

4.2 Secondary outcomes

Other pharmacodynamic measures:

1. Latency (Onset time 5): elapsed time to reduce the response of T1 to 5% of the initial contraction force after the infusion of cisatracurium.
2. Clinical Duration (DURATION 25%): the elapsed time for T1 recovery = 25% (Dur25%) of the original value of T1 after the infusion of cisatracurium.
3. Recovery Index (DURATION 25%-75%): the elapsed time between the T recovery=25% and 75% (Dur75%) after cisatracurium infusion.
4. Total Duration (Duration 95%): the elapsed time for T1 recovery of the response to reach 95% of the initial value after cisatracurium infusion.

Hemodynamics

1. Mean Arterial Pressure (MAP) and Heart Rate (HR) during the Induction Period
   These measures will be obtained at the following time points: (1) when the patient arrived in the operating room, (2) immediately before induction, (3) immediately before infusion of the solutions, (4) immediately after infusion of the drugs, (5) immediately before tracheal intubation, and (6) one minute after the tracheal intubation.

2. Mean Arterial Pressure (MAP) and Heart Rate (HR) during the Maintenance Period
   These measures will be obtained every 15 min until complete neuromuscular recovery.

4.3 Safety outcomes

Adverse events

Any adverse events will be documented.

5. Populations and subgroups to be analyzed

5.1 Populations

All randomized study subjects. This will be seen as the primary population for the analysis.

5.2 Subgroups

Not applicable

6. Analyses

All outcomes will be presented using descriptive statistics, normally distributed data by the mean and standard deviation (SD), and skewed distributions by the median and interquartile range (IQR). The Shapiro-Wilk test will be used to assess normality. The pharmacodynamic variables, clinical and demographic characteristics will be compared between the groups via the Kruskal-Wallis test, followed by Dunn's multiple comparison test or one-way ANOVA followed by the Tukey multiple comparison test, or the chi-square test, where appropriate. The area under the curve (AUC) will compare the study groups' hemodynamic responses. A p-value <0.05 will be considered statistically significant for all outcome variables. GraphPrisma version 8.3 will be used for all statistical analyses.

The subsections below will describe analyses in addition to the descriptive statistics.

6.1 Primary outcome

The primary analysis will compare intervention groups on their mean change in NMB. The difference in time course to TOF=90% will be the dependent variable. Study subjects will be considered as random effects and treatment groups as fixed effects. The estimated difference in mean change from baseline and the corresponding 95 % confidence interval (CI) will be presented.

6.2 Secondary outcomes

Other characteristics of NMB and Hemodynamic changes will be analyzed using the same method as for the primary outcome, including usage of the baseline value for the actual factor as a covariate.

7. Missing data

Not applicable.
